# Nanoscale Zinc Oxide Particles for Improving the Physiological and Sanitary Quality of a Mexican Landrace of Red Maize

**DOI:** 10.3390/nano8040247

**Published:** 2018-04-17

**Authors:** Juan Estrada-Urbina, Alejandro Cruz-Alonso, Martha Santander-González, Abraham Méndez-Albores, Alma Vázquez-Durán

**Affiliations:** 1 Faculty of Agricultural and Environmental Sciences, Autonomous University of Guerrero (UAGRO), Iguala de la Independencia, Guerrero 40010, Mexico; estrada.urbina.j@gmail.com; 2 National Autonomous University of Mexico-Superior Studies Faculty at Cuautitlan (UNAM–FESC), Campus 4, Multidisciplinary Research Unit L14-Annex 1 (Materials Science and Technology), Cuautitlan Izcalli, State of Mexico 54714, Mexico; sluckdmc@gmail.com (A.C.-A.); marsantander@gmail.com (M.S.-G.)

**Keywords:** ZnO nanoparticles, native maize seed, physiological and sanitary quality

## Abstract

In this research, quasi-spherical-shaped zinc oxide nanoparticles (ZnO NPs) were synthesized by a simple cost-competitive aqueous precipitation method. The engineered NPs were characterized using several validation methodologies: UV–Vis spectroscopy, diffuse reflection UV–Vis, spectrofluorometry, transmission electron microscopy (TEM), nanoparticle tracking analysis (NTA), and Fourier transform infrared (FTIR) spectroscopy with attenuated total reflection (ATR). A procedure was established to coat a landrace of red maize using gelatinized maize starch. Each maize seed was treated with 0.16 mg ZnO NPs (~7.7 × 10^9^ particles). The standard germination (SG) and accelerated aging (AA) tests indicated that ZnO NP-treated maize seeds presented better physiological quality (higher percentage of normal seedlings) and sanitary quality (lower percentage of seeds contaminated by microorganisms) as compared to controls. The application of ZnO NPs also improved seedling vigor, correlated to shoot length, shoot diameter, root length, and number of secondary roots. Furthermore, shoots and roots of the ZnO NP-treated maize seeds showed a marked increment in the main active FTIR band areas, most notably for the vibrations associated with peptide-protein, lipid, lignin, polysaccharide, hemicellulose, cellulose, and carbohydrate. From these results, it is concluded that ZnO NPs have potential for applications in peasant agriculture to improve the quality of small-scale farmers’ seeds and, as a result, preserve germplasm resources.

## 1. Introduction

Mexico has been the source of many foods that are now enjoyed by humans all over the world, such as many varieties of maize (*Zea mays* L.), bean (*Phaseolus vulgaris*), green tomato (*Physalis ixocarpa*), avocado (*Persea americana*), pineapple (*Ananas comosus*), guava (*Psidium guajava*), cinnamon (*Cinnamomum verum*), chili (*Capsicum annuum*), and cacao (*Theobroma cacao*), among others [1]. Mexico is considered the center of origin and diversification for maize, with a great diversity of native landraces [2]. Nowadays, the diversity of native cultivated maize landraces is diminishing, because of hybrid seed production and the introduction of improved varieties [3]. In recent times, native pigmented maize has received increased attention from a nutraceutical perspective, since phytochemicals—total phenolics, anthocyanins, ferulic acid, and carotenoids—have been found in considerable quantities [4,5].

In Mexico, maize is harvested throughout the year under diverse climatic conditions, from sea level to the highlands and from very low to very high humidity. In 2017, the total estimated production was over 21.5 million metric tons [6]. The state of Mexico is the second largest producer of maize, and also has the largest number of producers, 3.2 million, a high number considering that there are only 4 million in the entire country [7]. Traditional landraces of maize are still cultivated in small peasant holdings, using traditional rain-fed agrotechnology and usually multi-cropping systems known as “*milpa*” [8].

Seed is an essential resource for agriculture, and probably the most important in farming systems. Seed is also a determinant of yield and therefore productivity [9]. Ensuring that it is affordable and has good physiological and sanitary quality is a challenge for farmers, and a concern for society and the government, in order to ensure food security and self-sufficiency. The production system of native seeds, at medium and small scale, in developing countries such as Mexico continues to be artisanal motivating the development of technologies to improve production, benefit small-scale producers, and preserve the germplasm. For these reasons, the application of new products and technologies to improve the physiological and sanitary quality of maize seeds is an area of opportunity. In recent times, nanotechnology has allowed the generation of nanometer-scale materials, stimulating researchers to further study its possible application in agriculture. Zinc is one of the essential micronutrients required for the growth of plants, and the only metal represented in all six enzyme classes, hydrolases, transferases, oxidoreductases, isomerases, ligases, and lyases [10]. Zinc is also necessary for chlorophyll production, pollen functionality, fertilization, and germination, and also plays an important role in biomass production. It is well known that bulk zinc oxide (bZnO) is highly insoluble; however, by decreasing the size of the particle at the nanoscale level, the surface/volume ratio increases substantially, making it more soluble and bioavailable. Various studies reported both positive and negative effects of nanoparticles on higher plants; however, the use of zinc oxide nanoparticles (ZnO NPs) to improve the physiological and sanitary quality of pigmented native maize seeds and seedling vigor has not yet been reported. Consequently, the present research aims to: (1) synthesize and characterize ZnO NPs and apply them to native maize seeds in order to improve their physiological and sanitary quality, and (2) evaluate some seedling traits such as shoot length, shoot diameter, radical length, and number of secondary roots as measures of seedling vigor.

## 2. Materials and Methods 

### 2.1. Chemicals and Reagents

All the chemicals and reagents used in the experiment were of analytical grade and were used without further purification. Zinc nitrate hexahydrate (Zn(NO_3_)_2_·6H_2_O; 98% purity; CAS number 10196-18-6), sodium hydroxide (NaOH; ≥97% purity; CAS number 1310-73-2) and glycerol (C_3_H_8_O_3_; ≥99.5% purity; CAS number 56-81-5) were obtained from Sigma-Aldrich Co. (St. Louis, MO, USA). Food-grade maize starch was procured from Ingredion Mexico S.A. de C.V. (Tlalnepantla, Estado de Mexico, Mexico). Deionized water was used for preparing solutions.

### 2.2. Synthesis Protocol

ZnO NPs were synthesized by the precipitation method using Zn(NO_3_)_2_·6H_2_O and NaOH as precursors. Briefly, to an aqueous solution of Zn(NO_3_)_2_·6H_2_O (0.1 M), NaOH (1.5 M) solution was added slowly, drop by drop, from the sidewalls of the container to a pH value of 9, which resulted in the formation of a white suspension. The reaction was allowed to proceed for 2 h at room temperature under vigorous stirring after the alkaline solution was added completely. Afterwards, the solution was allowed to settle down for 24 h and then centrifuged (3200× *g*, 5 min) to remove the byproducts. The precipitate obtained was thoroughly washed with deionized water, dried in a convection oven (Binder model RE-115, Tuttlingen, Germany) at 120 °C for 2 h, and finally calcinated (Vulcan model A550, Woodbridge, Ontario, Canada) in air atmosphere at 600 °C for 4 h.

### 2.3. ZnO NP Characterization

#### 2.3.1. UV–Vis

Spectral analysis was done using a Cary 8454 UV–Vis Diode Array System spectrophotometer (Agilent Technologies, Santa Clara, CA, USA). Ultraviolet spectra were collected in the range of 200–800 nm at room temperature in 1 cm path quartz cell, and the absorbance at 376 nm, which is the characteristic absorption peak for wurzite hexagonal pure ZnO, was registered. 

#### 2.3.2. Optical Absorption Properties

Diffuse reflectance measurements were performed on a Lambda 365 UV–Vis spectrophotometer (Perkin Elmer, Waltham, MA, USA) equipped with an integrating sphere. Barium sulfate (BaSO_4_) was used as a reference. Spectra were recorded in the wavelength range of 300–700 nm in diffuse reflectance mode and subsequently transformed into the absorbance coefficient by the Kubelka–Munk function [11]. The band gap value was obtained from the plot of the Kubelka–Munk function versus the energy of the absorbed light [11,12].

#### 2.3.3. Fluorescence

The fluorescence spectra of the ZnO NPs aqueous suspension were measured using a fluorescence LS-55 spectrophotometer (Perkin Elmer, Waltham, MA, USA). Spectra were recorded in the wavelength range of 350–600 nm using 1 cm path quartz cell. The fluorescence spectrum was collected at an excitation wavelength of 325 nm using equally wide excitation and emission slits (10 nm).

#### 2.3.4. Transmission Electron Microscopy

The morphology of the ZnO NPs was examined using a highly integrated compact JEM-1010 transmission electron microscope (TEM; JEOL, Peabody, MA, USA) operated at an accelerating voltage of 80 kV. The high-contrast TEM is equipped with a 2k × 2k AMT CCD camera for digital image acquisition. TEM grids were prepared by placing a drop (10 µL) of the ZnO NP solution on carbon-coated copper grids, dried at room temperature.

#### 2.3.5. Nanoparticle Tracking Analysis

To determine particle size and particle concentration, a NanoSight NS300 (Malvern Instruments, Worcestershire, UK) equipped with a 532 nm green laser module was employed. NanoSight NTA software version 3.2.16 (Malvern Instruments, Worcestershire, UK) was used for data acquisition and analysis. Data were recorded using a 20× objective and a 60 s video clip. The Stokes–Einstein equation was used to calculate the mean hydrodynamic diameter. Three 60 s video measurements were recorded to provide the average mean and mode values of the ZnO NPs. Samples were prepared at an appropriate dilution (0.016 mg/mL), leading to a concentration between 10^6^ and 10^9^ particles, and were injected into the sample chamber, which had a volume of 0.3 mL. The capture setting parameters used were as follows: camera type: super-high-sensitivity complementary metal oxide semiconductor camera; camera level: 9; slider shutter: 607; slider gain: 15; number of frames: 1498; temperature: 21.1 °C; and viscosity: (water) 1.0 cP. 

#### 2.3.6. Fourier Transform Infrared Spectroscopy with Attenuated Total Reflection

ZnO NPs were further characterized using a Frontier SP8000 FTIR spectrophotometer (Perkin Elmer, Waltham, MA, USA) equipped with a deuterated triglycine sulfate detector and controlled with Spectrum 10.4.2 software (Perkin Elmer Ltd, Bucks, UK). Briefly, the ground samples (25 mg) were placed on top of the attenuated total reflection (ATR) crystal, and spectra were collected in the range of 350–4000 cm^−1^ at a resolution of 4 cm^−1^ by co-adding 32 scans. A background spectrum was obtained against air every day during the experiment. The spectra were collected in transmittance mode in quadruplicate, and the average value was used. 

### 2.4. Laboratory Experiments

#### 2.4.1. Maize Seed

Native red maize seed (Tlalnepantla-0917), grown and harvested in 2017 at Coyotepec (2308 m above mean sea level), was provided by the Peasant Producers of Seeds of the State of Mexico. The maize had a moisture content (MC) of 11%, and a thousand-kernel weight and test weight of 341.8 ± 0.22 g and 75.85 ± 0.45 kg/hL, respectively. The MC was determined by the forced air oven method drying at 103 °C for 72 h, with percentages calculated on a wet-weight basis. The seeds used were of uniform size to minimize errors in seed germination and seedling vigor tests. 

#### 2.4.2. Seed Conditioning with ZnO NPs

Seeds were surface-disinfected for 2 min in a 1% sodium hypochlorite solution and then rinsed 3 times with sterile deionized water. Maize seeds were coated with a starch paste formed by gelatinizing food-grade maize starch (1.5% *w*/*w*) in deionized water. Glycerol (1% *w*/*w*) was added as a plasticizer. When the temperature of the starch suspension was around 40 °C, 1.6 g of ZnO NPs was added (1600 ppm). The maize seeds were immersed in the coating suspension for 10 min, air-dried, and separated manually using a wire mesh. Under these conditions, each seed was coated with approximately 0.1 g of the starch suspension (equivalent to 0.16 mg ZnO NPs per seed). Controls comprised both uncoated and starch-coated seeds free of ZnO NPs.

#### 2.4.3. Standard Germination and Accelerated Aging Tests

Germination was carried out using 4 replications of 50 seeds on moistened crepe cellulose rolled paper. Treatments were randomized, and rolls were incubated in a growth chamber with an alternating photoperiod of 4 h at 25 °C. Seedlings were evaluated after 7 days according to the procedures of the Association of Official Seed Analysts [13]. For the accelerated aging (AA) test, seeds were placed in trays inside a plastic chamber containing 1 L of tap water (internal chamber), which was placed inside an AA chamber (external chamber). The seeds were aged at 41 °C and 100% relative humidity for 96 h. Following AA treatment, seeds were subjected to the standard germination (SG) test. In both tests, the results documented were the percentage of normal seedlings (physiological quality) and percentage of contamination by microorganisms such as bacteria and fungi (sanitary quality). Seedling vigor was correlated to shoot length, shoot diameter, root length, and number of secondary roots. Finally, the 7-day-old dry tissues from both roots and shoots were powdered and analyzed using FTIR-ATR spectroscopy as previously stated (spectra were collected in the range of 400–4000 cm^−1^). The peak areas of integration of the principal IR bands were calculated using Spectrum 10.4.2 software.

### 2.5. Experimental Design and Statistical Analysis

The experiment was conducted as a completely randomized design with 3 replicates. The Kruskal–Wallis nonparametric test was performed to assess the physiological and sanitary quality of the maize seeds. Means comparison for seedling vigor parameters was carried out utilizing the Tukey test with SAS software (SAS Institute Inc. Cary, NC, USA) [14]. A significance value of α = 0.05 was used to distinguish significant differences between treatments.

## 3. Results and Discussion

### 3.1. ZnO NP Characterization

#### 3.1.1. Optical Properties

**Figure 1:**
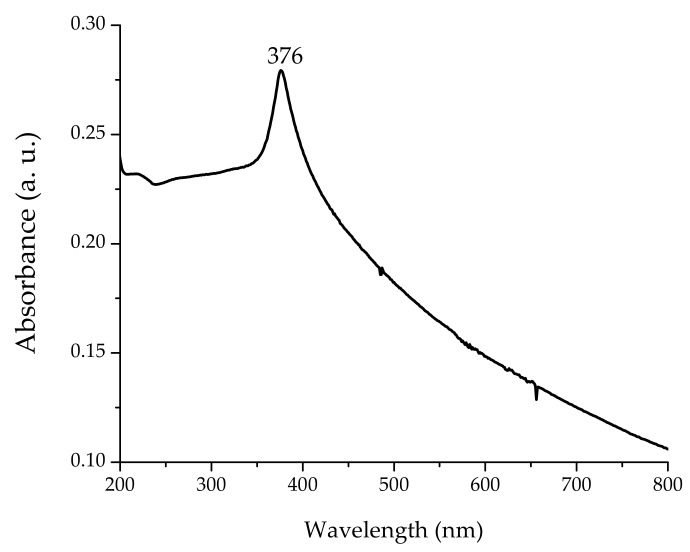
Representative UV–Vis absorption spectrum of synthesized zinc oxide nanoparticles (ZnO NPs).

UV–visible absorption spectroscopy is widely used as a technique to examine the optical properties of certain nanoscale particles. The absorption spectrum of ZnO NPs is depicted in Figure 1. The spectrum reveals the characteristic absorption peak at 376 nm (3.30 eV), which can be assigned to the intrinsic band-gap absorption of ZnO [15]. This sharp peak indicates the monodispersed nature of the nanoparticle distribution and that most of the particles are in nanoscale. No other peaks were observed in the spectrum, which confirms that the synthesized product is only ZnO (the wurzite hexagonal phase of ZnO). 

**Figure 2:**
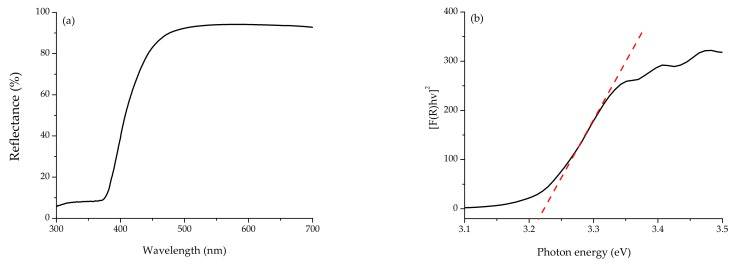
(**a**) Diffuse reflectance spectra, and (**b**) Kubelka–Munk transformed reflectance spectra of synthesized ZnO nanoparticles.

To determine the optical band gap of synthesized ZnO NPs, their reflectance spectra were measured (Figure 2a). As seen in Figure 2, the reflectance spectra show a strong decrease around 450 nm. This decrease is related to the electron transitions occurring in the optical band gap. In order to determine the precise value of the band gap, the reflectance values were converted to absorbance by applying the Kubelka–Munk function [16]. As a result, the direct band gap of ZnO NPs was estimated from the plot of (F(R)hʋ)^2^ versus the photon energy (hʋ), and the value was 3.21 eV (Figure 2b). Ashar et al. [17] reported band gap values of 3.37 eV for bZnO and 3.24 eV for ZnO pseudo-sphere particles obtained by the precipitation route. This slight variation in band gap values could be attributed to the different levels of oxygen vacancies in the ZnO NPs [18].

**Figure 3:**
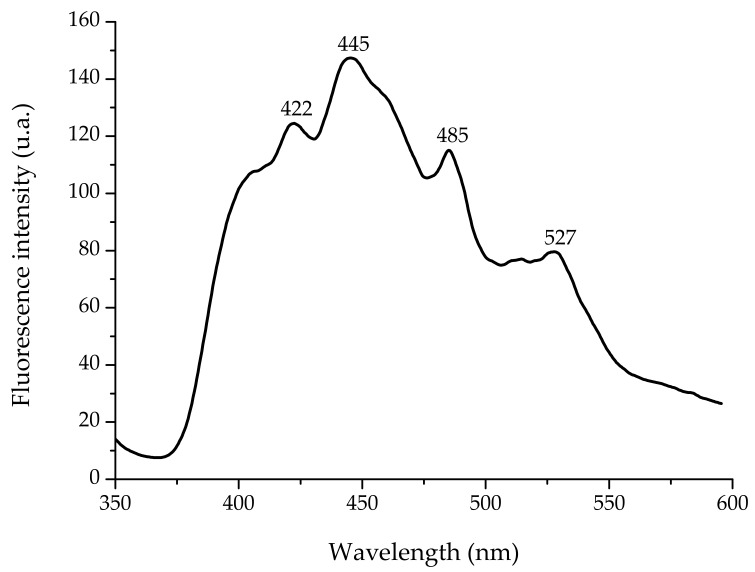
Fluorescence emission spectrum of synthesized ZnO nanoparticles. The excitation wavelength was 325 nm.

The fluorescence spectrum of the ZnO NP aqueous suspension at an excitation wavelength of 325 nm is shown in Figure 3. The spectrum exhibits all the characteristic emission peaks (422 nm, 445 nm, 485 nm, and 527 nm) of pure nano-ZnO. In this research, the blue emission peak at 422 nm is attributed to the formation of Zn interstitial defects, the strong blue emission at 445 nm and the weak blue-green emission at 485 nm are attributed to the formation of oxygen vacancy defects, and the green fluorescence at 527 nm may have originated from the antisite defects. It is well known that visible luminescence is mainly due to defects related to deep-level emission, such as Zn interstitials and oxygen vacancies [19]. Generally, the single ionized oxygen vacancy is responsible for the green emission in ZnO, which results from the recombination of a photo-generated hole with the single ionized charge state of this defect [20].

#### 3.1.2. Morphology of ZnO NPs

**Figure 4:**
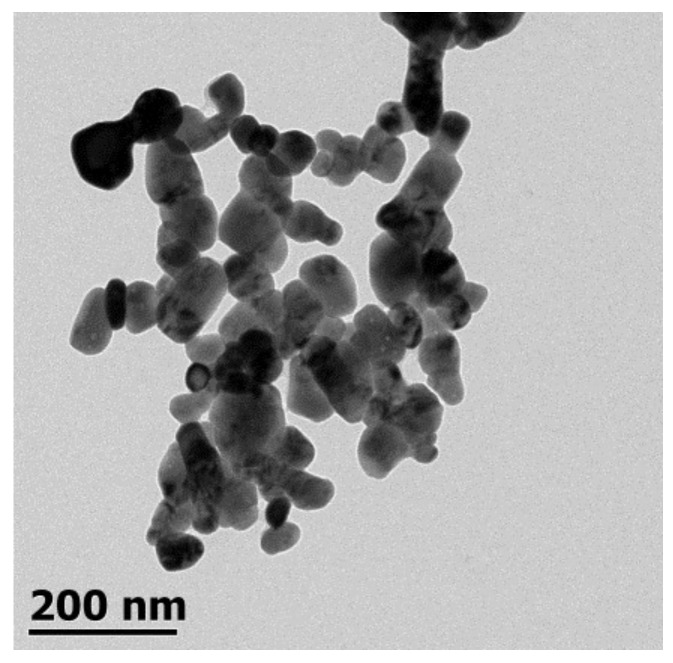
Illustrative transmission electron microscopy (TEM) image of synthesized ZnO nanoparticles.

Figure 4 shows the TEM micrograph of synthesized ZnO NPs. In general, the image reveals that most of the particles have a quasi-spherical shape, with diameters in the range of 30–125 nm. Such a wide range of particle sizes may be a result of the time-dependent nucleation and growth of nanoparticles during synthesis. Brayner et al. [21] stated that one of the factors that influences the size and shape of ZnO materials is the hydrolysis ratio. Figure 4 also shows that some agglomeration occurred, which could be attributed to the large specific surface area and high surface energy of the particles [22].

#### 3.1.3. Particle Size and Particle Concentration Using NTA

**Figure 5:**
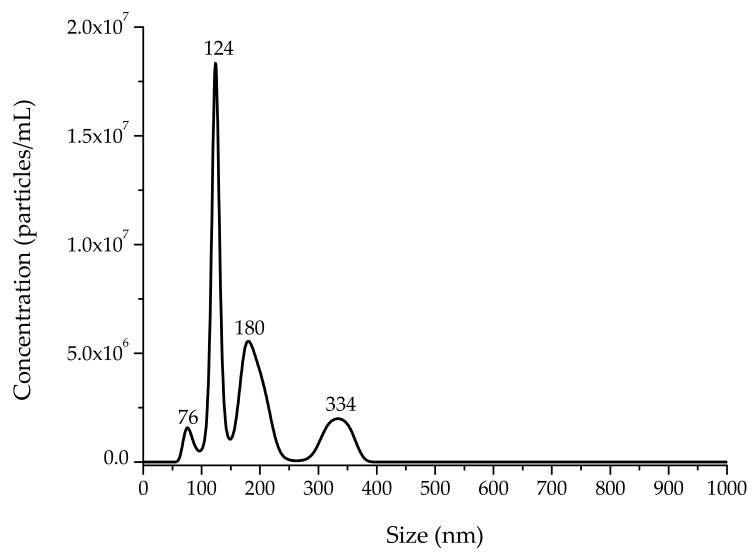
Particle number concentration and particle size distribution of synthesized ZnO nanoparticles.

The particle size distribution of synthesized ZnO NPs is shown in Figure 5. The TEM technique yielded a fairly narrow size distribution (30–125 nm), while the NTA indicates a relatively broad size distribution (76–334 nm). The mean and mode particle sizes calculated from the NTA were 180 nm and 124 nm, respectively. Figure 4 shows a nonparametric distribution of the ZnO NPs, with the majority ranging around 124 nm in size, as well as two other minor populations with bigger diameters (180 nm and 334 nm). The particles with large hydrodynamic diameter could be due to the formation of aggregates from primary particles in the suspension [23]. Regarding particle concentration, the ZnO NP preparation dropped 7.7 × 10^8^ particles per mL. Consequently, each maize seed picked up approximately 7.7 × 10^9^ particles.

#### 3.1.4. FTIR-ATR Analysis

**Figure 6:**
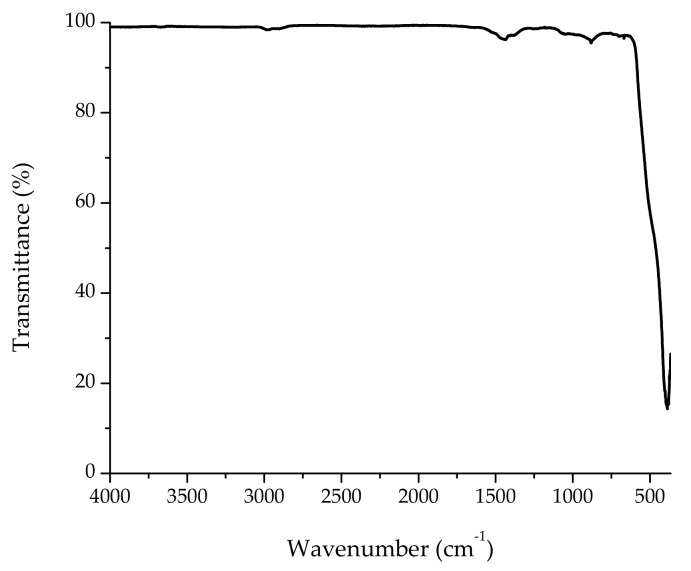
Typical Fourier transform infrared spectroscopy with attenuated total reflection (FTIR-ATR) spectrum of synthesized ZnO nanoparticles.

The FTIR spectrum of ZnO NPs calcinated at 600 °C can be seen in Figure 6. As a result of the calcination process, the bands in the region of 2800–3600 cm^−1^ were lost due to the removal of water molecules. However, a broad band was observed at around 391 cm^−1^ which was attributed to the Zn–O vibration mode [15]. There were other insignificant bands at 1440 cm^−1^, 1377 cm^−1^, and 882 cm^−1^ in the spectrum; these absorption bands were likely related to CO_2_ absorbed from the air atmosphere and can therefore be neglected.

### 3.2. Maize Seed Coating Studies

#### 3.2.1. Physiological and Sanitary Quality of Maize Seeds 

The effects of ZnO NPs on the physiological and sanitary quality of maize seeds are shown in Table 1. In general, during SG and AA tests, better germination percentages (97% and 90%) due to the ZnO NP coating were observed as compared to control groups. In this context, Adhikari et al. [24] reported enhanced germination percentages in maize (93 to 100%) due to nanoscale (<100 nm) ZnO coating at 25 mg Zn/g seed as compared to uncoated seeds (80%). Zhang et al. [25] also found better germination percentages for maize seeds soaked in ZnO NPs suspensions containing 10, 100, or 1000 mg ZnO/L. Similarly, Subbaiah et al. [26] observed a higher germination percentage (80%) in maize seeds treated with 1500 ppm of ZnO nanoparticulates compared to controls (35 to 40%). Data shown by these researchers are consistent with our results.

The AA test is recommended to measure maize seed vigor. In this research, treating aging ZnO NP seeds for up to 96 h at 41 °C caused a moderate reduction in the germination percentage (90%); however, a significant reduction in germination occurred after 96 h for uncoated seeds (68%). In addition, starch-coated seeds significantly preserved the germination rate (80%) during the AA test (Table 1). These results indicate that the treatment of maize seeds with ZnO NPs has a double role during germination: (1) the starch coating serves as a protective film for the seeds, decreasing their respiration, to avoid an advanced stage of deterioration, and (2) zinc protects both the integrity and permeability of the cell membrane, acting as a structural stabilizer for proteins and DNA-binding proteins [27,28], thus improving the tolerance of the seeds to the abiotic stress conditions. 

Regarding sanitary quality, low percentages of maize seed infection during SG (37%) and AA (8%) tests were observed in the ZnO NP treatment (Table 1). Uncoated seeds presented vigorous microbiological contamination, mainly by fungi from three different genera (*Fusarium* sp., *Penicillium* sp., and *Trichoderma* sp.). It has been well-established that ZnO NP coating inhibits bacterial and fungal infection of different seeds such as maize, soybean (*Glycine max* L.), pigeon pea (*Cajanas cajan* L.), and ladies’ finger (*Abelmoschus esculentus* L.) at the time of germination [24]. He et al. [29] evaluated the antifungal activity of ZnO NPs (70 ± 15 nm) and their mode of action against two pathogenic fungi (*Botrytis cinerea* and *Penicillium expansum*). Results showed that ZnO NPs significantly inhibit the growth of *B. cinerea* by affecting cellular functions. In contrast, ZnO NPs prevented the development of conidiophores and conidia of *P. expansum*, which eventually caused morphological alterations in hyphae that led to hyphae death. The results of this study go along with the principles mentioned above.

#### 3.2.2. Maize Seedling Vigor Evaluation

In general, ZnO NPs significantly improved maize seedling vigor compared to the control (uncoated seeds). Greater shoot length (14.4 mm and 11.1 mm) and shoot diameter (3.4 mm and 2.0 mm) were observed in seeds during SG and AA tests. Additionally, significantly greater root elongation and an increased number of secondary roots were also observed in ZnO NP-treated seeds (Table 2). This effect can be related to Zn’s activity as a precursor in the production of auxins, a group of growth regulators that promote elongation and cell division [30]. Auxin transport and auxin signaling pathways also have an important role in lateral-root formation [31]. Salama [32] reported that an Ag NP concentration of 20 to 60 ppm showed statistically significant stimulation of shoot and root elongation in common bean (*Phaseolus vulgaris* L.) and maize. Morales et al. [33] found significantly longer shoots and larger roots in cilantro (*Coriandrum sativum* L.) plants treated with 125 mg/kg of CeO_2_ NPs. Subbaiah et al. [26] reported a high seedling vigor index, calculated using the formula reported by Abdul-Baki and Anderson [34], in maize treated with 1500 ppm of nanoscale ZnO particulates prepared with a modified oxalate decomposition methodology. In another study with peanuts (*Arachis hypogaea* L.), ZnO NPs (1000 ppm) showed positive effects on seedling vigor indices [35]. These findings are in close agreement with the results found in this research.

#### 3.2.3. FTIR-ATR Studies (Shoots and Roots)

To identify any changes in the specific functional groups in maize seedlings, FTIR-ATR studies were conducted on the seven-day-old dry tissues from both shoots and roots. Table 3 shows the most significant FTIR bands and their corresponding assignations. In addition, an FTIR spectra comparison is depicted in Figure S1, and the equivalent integration areas are shown in Table 4. In general, significant differences in band areas were noted among treatments. ZnO NP-treated seeds showed a marked increment in almost all band areas in shoot and root FTIR spectra, most notably in the peptide-protein, lipid, lignin, polysaccharide, hemicellulose, cellulose, and carbohydrate bands. Morales et al. [33] reported increments in the amide (1550 cm^−1^) and lignin (1515 cm^−1^) areas in shoots and augments in the lipid (2840–2960 cm^−1^) and carbohydrate (900–1200 cm^−1^) areas in roots of cilantro treated with CeO_2_ NPs, which is consistent with our findings.

## 4. Conclusions

In summary, quasi-spherical-shaped ZnO NPs were synthesized by a simple cost-competitive aqueous precipitation method and successfully used to improve the germination rate and vigor of native pigmented maize seeds. However, further studies are required to determine whether the ZnO NP treatment currently applied would continue to sustain the viability and vigor of maize seeds when they are subjected to rural storage techniques, since peasant producers completely lack access to postharvest technology, including the most basic storage structures and technical assistance to help store their native maize seeds. Research in this direction is in progress.

## Figures and Tables

**Table 1:** Effect of ZnO NPs on the physiological and sanitary quality of maize seeds during standard germination and accelerated aging tests.

Treatment	Germination (%)		Contamination (%)	
	SG	AA	SG	AA
Uncoated	80 ± 3 ^a^	68 ± 4 ^a^	70 ± 4 ^a^	45 ± 5 ^a^
Starch-coated	83 ± 2 ^a^	80 ± 2 ^b^	58 ± 3 ^ab^	40 ± 4 ^a^
ZnO NPs	97 ± 2 ^b^	90 ± 3 ^c^	37 ± 4 ^c^	8 ± 2 ^b^

Means with different letter in the same column are statistically different (Kruskal–Wallis *p* < 0.05). Mean value ± standard error. SG = Standard Germination; AA = Accelerated Aging.

**Table 2:** Effect of ZnO NPs on maize seedling vigor during standard germination and accelerated aging tests.

	Treatment	Length (cm)		Diameter (mm)	
		SG	AA	SG	AA
Shoot	Uncoated	12.8 ± 0.4 ^a^	8.1 ± 0.5 ^a^	2.8 ± 0.1 ^a^	1.6 ± 0.1 ^a^
	Starch-coated	13.5 ± 0.5 ^ab^	9.6 ± 0.5 ^a^	3.1 ± 0.1 ^b^	1.7 ± 0.2 ^a^
	ZnO NPs	14.4 ± 0.5 ^b^	11.1 ± 0.6 ^b^	3.4 ± 0.1 ^c^	2.0 ± 0.1 ^b^
		**Length (cm)**		**Secondary Roots (Number)**	
Root	Uncoated	16.2 ± 0.7 ^a^	14.9 ± 0.6 ^a^	2.6 ± 0.1 ^a^	1.6 ± 0.1 ^a^
	Starch-coated	20.3 ± 0.7 ^b^	16.6 ± 0.5 ^b^	3.3 ± 0.2 ^b^	2.9 ± 0.2 ^b^
	ZnO NPs	20.4 ± 0.7 ^b^	17.9 ± 0.6 ^b^	3.4 ± 0.2 ^b^	3.0 ± 0.2 ^b^

Means with different letter in the same column are statistically different (Tukey *p* < 0.05). Average value ± standard error. SG = Standard germination; AA = Accelerated aging.

**Table 3:** Band assignments of the main active vibrations present in the FTIR-ATR spectra of maize shoots and roots.

Band	Wavenumber (cm^−1^)						Functional Group and Commonly Assigned Component
	Shoots			Roots			
	Uncoated	Starch-Coated	ZnO NPs	Uncoated	Starch-Coated	ZnO NPs	
A	3127	3189	3244	3168	3190	3253	N–H stretching vibrations (peptide and protein).
B	-	2915	2920	2926	2926	2920	C–H symmetric/asymmetric stretch (lipid).
C	2359	2354	2366	2337	2343	2341	N≡N stretch in primary amines.
D	-	1627	1630	1579	1579	1582	Aromatic C=C stretch (lignin).
E	-	-	1410	1375	1364	1367	C–H bends from symmetric –(CH_3_)n– –(CH_2_)n– (lipid, polysaccharide and cellulose).
F	-	-	1243	-	1250	1249	C–O–H deformation asymmetric (hemicellulose and cellulose).
G	-	1036	1032	1033	1034	1037	C–O stretching/C–O bending of the C–O–H carbohydrate.

**Table 4:** FTIR-ATR band areas of maize shoots and roots.

Band	Band Area (Area Units)					
	Shoots			Roots		
	Uncoated	Starch-Coated	ZnO NPs	Uncoated	Starch-Coated	ZnO NPs
A	103.74	302.16	636.94	249.2	313	512.75
B	0	17.3	107.53	9.87	8.79	36.65
C	172.35	138.28	151.72	202.94	209.46	229.93
D	87.73	146.52	349.66	89.51	102.36	136.33
E	0	9.79	124.54	14.42	23.52	90.3
F	0	0	43.37	0	8.25	20.47
G	21.16	384.55	1139.9	308.41	466	714.11

## Supplementary Materials

The following are available online at http://www.mdpi.com/2079-4991/8/4/247/s1, Figure S1: Comparative FTIR spectra of (a) maize seedling shoot, and (b) root tissues.
