# Supplementary figures and images for: Nanoscale Zinc Oxide Particles for Improving the Physiological and Sanitary Quality of a Mexican Landrace of Red Maize
Source: Nanomaterials (Basel). 2018 Apr 17;8(4):247. doi: 10.3390/nano8040247 (PMC5923577; doi:10.3390/nano8040247)

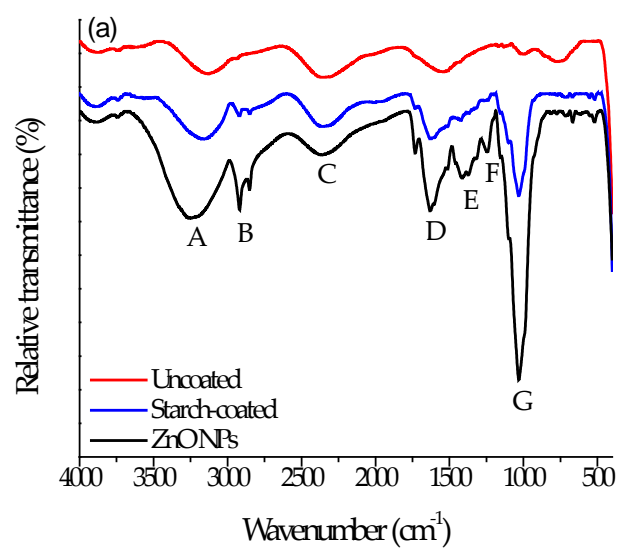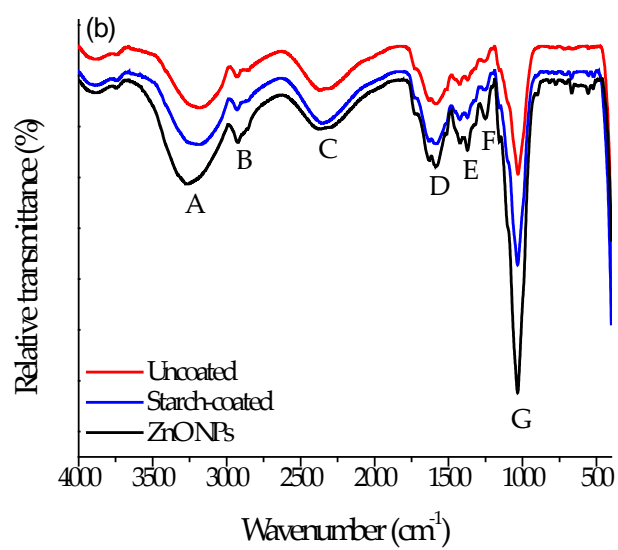

**Figure S1.** Comparative FTIR spectra of (a) maize seedling shoot, and (b) root tissues.

Supplement: Supplementary file 1 [file nanomaterials-08-00247-s001.pdf]
